# Modeling HIV quasispecies evolutionary dynamics

**DOI:** 10.1186/1471-2148-7-S2-S5

**Published:** 2007-08-16

**Authors:** Luca Sguanci, Franco Bagnoli, Pietro Liò

**Affiliations:** 1 CSDC, Center for the Study of Complex Systems, Firenze, Italy; 2 Computer Laboratory, University of Cambridge, CB3 0FD Cambridge, UK; 3 Dipartimento di Energetica, Università di Firenze, Via S. Marta 3, 50139 Firenze, Italy; 4 INFN, sezione di Firenze, Italy

## Abstract

**Background:**

During the HIV infection several quasispecies of the virus arise, which are able to use different coreceptors, in particular the CCR5 and CXCR4 coreceptors (R5 and X4 phenotypes, respectively). The switch in coreceptor usage has been correlated with a faster progression of the disease to the AIDS phase. As several pharmaceutical companies are starting large phase III trials for R5 and X4 drugs, models are needed to predict the co-evolutionary and competitive dynamics of virus strains.

**Results:**

We present a model of HIV early infection which describes the dynamics of R5 quasispecies and a model of HIV late infection which describes the R5 to X4 switch. We report the following findings: after superinfection (multiple infections at different times) or coinfection (simultaneous infection by different strains), quasispecies dynamics has time scales of several months and becomes even slower at low number of CD4+ T cells. Phylogenetic inference of chemokine receptors suggests that viral mutational pathway may generate a large variety of R5 variants able to interact with chemokine receptors different from CXCR4. The decrease of CD4+ T cells, during AIDS late stage, can be described taking into account the X4-related Tumor Necrosis Factor dynamics.

**Conclusion:**

The results of this study bridge the gap between the within-patient and the inter-patients (i.e. world-wide) evolutionary processes during HIV infection and may represent a framework relevant for modeling vaccination and therapy.

## Background

The relationship between phenotype and survival of the genotype is central to both genetics and evolution. Viruses represent a good sized-complexity phenotype to study and they show rapid evolution. Selection pressures mainly depend on the interaction strength with the receptor vital for the entry into the target cell. Human immunodeficiency virus type 1 (HIV-1) infection is characterized by the progressive loss of CD4+ T cells. Infection by most strains of HIV-1 requires interaction with CD4 and a chemokine receptor, either CXCR4 or CCR5. During early stages of HIV-1 infection, viral isolates most often use CCR5 to enter cells and are known as R5 HIV-1. Later in the course of HIV-1 infection, viruses that use CXCR4 in addition to CCR5 (R5X4) or CXCR4 alone (X4 variants) emerge in about 50% patients (switch virus patients) [1,2]. These strains are syncytium-inducing and are capable of infecting not only memory T lymphocytes but also naive CD4+ T cells and thymocytes through the CXCR4 coreceptor. The switch to using CXCR4 has been linked to an increased virulence and progression to AIDS, probably through the formation of cell syncytia and apoptosis of T cell precursors. X4 HIV strains are rarely, if ever, transmitted, even when the donor predominantly carries X4 virus. Clevestig [3] found that in children, the X4 virus developed from their own R5 population, and was not caused by transmission from the mother. CXCR4 is expressed on a majority of CD4+ T cells and thymocytes, whereas only about 5 to 25% of mature T cells and 1 to 5% of thymocytes express detectable levels of CCR5 on the cell surface [4]. It is noteworthy that X4 HIV strains stimulate the production of cellular factor called Tumor Necrosis Factor (TNF), which is associated with immune hyperstimulation, a state often implicated in T-cell depletion [5].

Tumor necrosis factor (TNF)-related apoptosis-inducing ligand (TRAIL) is produced mainly by monocytes; it is a type II transmembrane protein belonging to the TNF family, homologous to Fas ligand (FasL), which is a well-characterized apoptosis-inducing ligand. In particular the Fas-associated domain or a Fas-associated domain-like adaptor molecule leads to activation of the caspase cascade, resulting in apoptotic cell death. TNF seems able to both inhibit the replication of R5 HIV strains while having no effect on X4 HIV and to down regulate the number of CCR5 co-receptors that appear on the surface of T-cells [6]. Plasma TRAIL is elevated in HIV-1 infected patients and is decreased by Highly Active Anti Retroviral Therapy (HAART). Thus, when HAART decreased viral load, there is a concomitant decrease in plasma TRAIL, which may be one of the reasons for the efficacy of antiviral therapy [7].

### Mathematical modeling

The use of mathematical models is an insightful and essential complement to in vivo and in vitro experimental design and interpretation. Indeed mathematical models of HIV dynamics have proven valuable in understanding the mechanisms of many of the observed features of the progression of the HIV infection, see for example [8-15].

A powerful concept in understanding HIV variability and its consequences is that of quasispecies, accounting for the result of evolution not being the selection of a single sequence (genotype), rather a distribution of quasi-identical sequences, termed the *quasispecies*, centered around a master sequence. Quasispecies are the combined result of mutations and recombination, that originate variability, and of co-infection (simultaneous infection), superinfection (delayed secondary infection) and selection, that keep variability low. HIV-1-infected individuals show heterogeneous viral populations, best described as viral quasispecies [16]. Infact, the infection capacity of mutants may vary and also their speed of replication [17].

Moreover, since the number of targets (the substrate) is limited, fitter clones tend to eliminate less fit mutants, which are subsequently regenerated by the mutation mechanism. Mutations are a key ingredient for exploring the genetic space in the search for the fitness maximum, but are also responsible for the disappearance of the quasispecies when exceeding a critical mutation rate, the *error threshold *[18].

Taking into account state-of-the-art models of HIV infection, we address the issue of studying the coevolutive and competitive dynamics of different strains of HIV-1 virus also leading to the R5 to X4 phenotype switching. Ribeiro and colleagues [19] have recently presented a model of R5 to X4 switch based on the hypothesis that X4 and R5 viruses have a preferential tropism for naive and memory T cells, respectively. Here we prefer to follow the mutational hypothesis supported by several experiments [1-4]. In the next section we introduce two models: a quasispecies model for R5 phase in which several R5 strains appear by mutations, co-infection and superinfection. In the limit of a single quasispecies we are able to find the same values observed experimentally and in other models (most notably Perelson's standard model). We test the model in the scenarios of co-infection and superinfection using parameters derived from biological and medical literature. A second model focuses on the R5 to X4 shift and the hyperstimulation of T cell precursors through TNF. The results of the numerics reproduce well the decreasing dynamics of CD4+ after the appearance of X4 strains and make the model suitable for further investigations on antiretroviral therapies and their effects on disease progression. The R5 to X4 switch is also investigated by using phylogenetic models of the amino acid sequences of the human and mice chemokine receptor families. The analysis of the mutational pathway suggests that the switch from R5 to X4 may allow the HIV to bind to other chemokine receptors, thus likely leading to immune system signaling disfunctions.

## Results and discussion

### Modeling HIV dynamics and the immune response

The basic model of HIV-1 dynamics, first introduced by Perelson and colleagues in 1995 [8], is a class model considering three variables: uninfected cells, infected cells and free viruses. A system of three differential equations describes how these quantities change over time. In the basic model by Perelson uninfected cells encounter free virus and turn into infected cells. The rate of production of infected cells is proportional to the product of the density of uninfected cells times the density of free virions. Free virions are produced by infected cells. Uninfected cells, infected cells and free virions die at fixed rate. Uninfected cells are also assumed to be constantly replaced by the immune system. These assumption define the basic model of virus dynamics.

**Figure 1:**
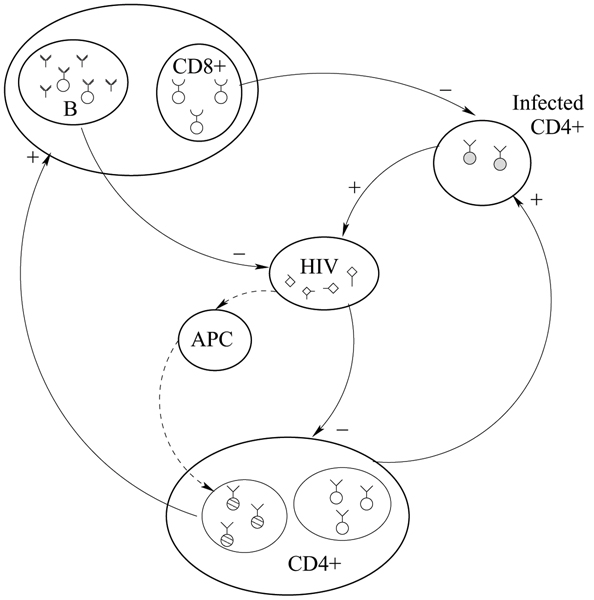
**Representation of the interactions between cells and/or cells and viruses**. Representation of the interactions between cells and/or cells and viruses. HIV strains infect CD4+ T cells that become infected and produce new viruses. At the same time HIV peptides are presented via APC cells to T-Helper cells that become activated. Activated CD4+ T cells trigger B and CD8+ cells reactions: the first release antibodies that bind to the antigen while the latter directly remove infected CD4+ T cells. '+' and '-' signs indicate cell/virus production or removal.

We extended this basic model of HIV-1 dynamics, by including the response by the immune system and the role of B lypmhocytes, see Figure 1. There are several works that discuss the role of B cells in the immune response [20]. Since the progression to AIDS has been found to correlate well with CD4+ T cells decrease, B cells are thought to play a minor role in the immune response to HIV. Note that B cells can act only as predator to the HIV, so their coupling with HIV dynamics is different from that of T cells. Our aim is to present a more general model framework of both T and B immune responses to HIV. We have first considered the following system of differential equations describing the dynamics of a single viral strain:

T˙
 MathType@MTEF@5@5@+=feaafiart1ev1aaatCvAUfKttLearuWrP9MDH5MBPbIqV92AaeXatLxBI9gBaebbnrfifHhDYfgasaacH8akY=wiFfYdH8Gipec8Eeeu0xXdbba9frFj0=OqFfea0dXdd9vqai=hGuQ8kuc9pgc9s8qqaq=dirpe0xb9q8qiLsFr0=vr0=vr0dc8meaabaqaciaacaGaaeqabaqabeGadaaakeaacuWGubavgaGaaaaa@2DE6@ = (*λ *+ *γ*^(*T*)^*IT*)(1 - *T*/*K*) -(*δ*_*T *_+ *βV*)*T*,

I˙
 MathType@MTEF@5@5@+=feaafiart1ev1aaatCvAUfKttLearuWrP9MDH5MBPbIqV92AaeXatLxBI9gBaebbnrfifHhDYfgasaacH8akY=wiFfYdH8Gipec8Eeeu0xXdbba9frFj0=OqFfea0dXdd9vqai=hGuQ8kuc9pgc9s8qqaq=dirpe0xb9q8qiLsFr0=vr0=vr0dc8meaabaqaciaacaGaaeqabaqabeGadaaakeaacuWGjbqsgaGaaaaa@2DD0@ = *βVT *- (*δ*_*I *_+ *γ*^(*I*)^*T*)*I*,

V˙
 MathType@MTEF@5@5@+=feaafiart1ev1aaatCvAUfKttLearuWrP9MDH5MBPbIqV92AaeXatLxBI9gBaebbnrfifHhDYfgasaacH8akY=wiFfYdH8Gipec8Eeeu0xXdbba9frFj0=OqFfea0dXdd9vqai=hGuQ8kuc9pgc9s8qqaq=dirpe0xb9q8qiLsFr0=vr0=vr0dc8meaabaqaciaacaGaaeqabaqabeGadaaakeaacuWGwbGvgaGaaaaa@2DEA@ = *πI *- (*c *+ *γ*^(*V*)^*T*)*V*.

This model considers the T-helper (CD4+) cells (*T*) and HIV virus particles (*V*); the T cells can become infected (*I*). With respect to Refs. [8,9], in Eq. (1), we describe how the number of naive T cells (*T*) which have passed the thymus selection, depends on rate of formation in the bone marrow (*λ*) and on clonal amplification upon stimulation by infected cells, *I *(term *IT*). They decrease with a rate that is the sum of a natural clearance (*δ*_*T*_*T*) due to cell aging and cell destruction upon virus infection, (*VT*). The density of T cells is limited by a saturating density/lymphonode capacity factor, *K*. Following Ref. [14] we have set *K *= 10^12^.

The second equation describes the rate of infection, described by *β*, of naive T cells upon the interaction with the virus (term *VT*). Infected T cells are cleared out at a fixed rate, *δ*_*I*_, and due to the action of natural killer cells, CD8+ (term *TI*).

The third equation describes the budding of viruses from infected cells, *π*. Virus particles are cleared out at rate *c *(defective viruses) and after immunoglobulin binding and subsequent engulfments by the macrophages (term *TV*).

The *γ *parameters have the same meaning of the constant of association in chemistry, or can be thought as a combination of both the probability of interaction and the interaction strengths between cells (*γ*^(*T*)^, *γ*^(*I*)^) or between cells and viruses (*γ*^(*V*)^). Note that in the limit *γ *→ 0 and *K *→ ∞, we recover the pattern of the standard model [13].

The B cell response is modeled using the parameters corresponding to the T cells which activate them by receptor recognition. Here, we have assumed the immunoglobulin concentration, which represent the B cell response, to be linearly correlated to the concentration of activated B cells. These in turn are supposed linearly correlated to the concentration of the CD4+ T cells. Exploratory analysis with different parameters, suggested us that Eq. (3) allows to keep a minimum number of parameters without loss of important details.

### A quasispecies framework

A meaningful model to study the evolution of a viral population is that of quasispecies. The quasispecies concept was put forward by Manfred Eigen and Peter Schuster [21] within the framework of physical chemistry. Their model accounts for the description of the process of the Darwinian evolution of self-replicating entities and shows that the result of evolution is not the selection of a single sequence, rather a distribution of sequences centered around a master sequence. The quasispecies behavior of viruses is nowadays recognised as a key element for the understanding and modeling of viral evolution and disease control [22]. The extreme mutation rate of RNA genetic elements, together with their large population sizes, endows them with the characteristics of quasispecies populations. Most progeny genomes of individual (clonal) RNA viruses generally differ from one another in having one or more mutations scattered randomly along their genomes; thus comprising a quasispecies cloud or swarm of related, but not identical variants.

A quasispecies model of HIV infection can be derived by the model previously introduced. We adopt a quasispecies description of the process by first hypothesizing the evolution of the virus to be characterized by only one slight changing phenotypic trait, e.g. the shape of the envelope and its corresponding binding affinity. This assumption is justified if the phenotypes are determined by few viral protein functional determinants which are both independent and differ only few DNA bases, i.e. few mutations can change one determinant into another. This corresponds to map the different phenotypes of the virus on a linear space (see [23,24] for similar assumptions). In fact this binding does not need to be a perfect match, but has a certain degree of tolerance. Similar phenotypes compete for the same coreceptor and mutations can be the key factor to give origin to a new phenotype able to use a different coreceptor. On the other hand T lymphocytes look forward to kill infected T cells and free virions. Their recognition ability of viral antigens depends on the binding between a T cell receptor (TCR) and a viral epitope.

Based upon the above schematization, the population of T cells can be thought of as composed by different phenotypes corresponding to the different TCRs and the virus populations as characterized by a specific binding ability. Thus the clonal amplification of naïve T cells (of population *i*) depends on the ability of (*i*^th ^class) T cell to recognize all the infected T cells (carrying an epitope from the *k*^th ^class of viruses). The rate of infection of naïve T cells (of class *i*) depends on all the viruses (containing the epitope *k*). In the same way, the clonal expansion of infected T cells (of class *k*, meaning that they have been infected by a virus of class *k *irrespective of the original T class) depends on the interaction of these viruses with all the T cells (of any class *i*).

It's noteworthy that this schematization is valid for the quasispecies case and for the undifferentiated one. In the latter case, the indication of strains (in parenthesis) should be neglected.

#### A model of the early R5 phase evolution

In someone who is newly infected by HIV, several variants of the virus, called R5, are often the only kind of virus that can be found. A meaningful way to model strain mutations, coinfection and superinfection is to extend the model previously introduced by incorporating multi strain evolutive dynamics. Thus the R5 quasispecies dynamics can be described by the following set of equations:

T˙i=(λi+∑kγik(T)IkTi)(1−1K∑iTi)−(δT+∑kβkVk)Ti,
 MathType@MTEF@5@5@+=feaafiart1ev1aaatCvAUfKttLearuWrP9MDH5MBPbIqV92AaeXatLxBI9gBaebbnrfifHhDYfgasaacH8akY=wiFfYdH8Gipec8Eeeu0xXdbba9frFj0=OqFfea0dXdd9vqai=hGuQ8kuc9pgc9s8qqaq=dirpe0xb9q8qiLsFr0=vr0=vr0dc8meaabaqaciaacaGaaeqabaqabeGadaaakeaacuWGubavgaGaamaaBaaaleaacqWGPbqAaeqaaOGaeyypa0ZaaeWaaeaaiiGacqWF7oaBdaWgaaWcbaGaemyAaKgabeaakiabgUcaRmaaqafabaGae83SdC2aa0baaSqaaiabdMgaPjabdUgaRbqaaiabcIcaOiabdsfaujabcMcaPaaakiabdMeajnaaBaaaleaacqWGRbWAaeqaaOGaemivaq1aaSbaaSqaaiabdMgaPbqabaaabaGaem4AaSgabeqdcqGHris5aaGccaGLOaGaayzkaaWaaeWaaeaacqaIXaqmcqGHsisldaWcaaqaaiabigdaXaqaaiabdUealbaadaaeqbqaaiabdsfaunaaBaaaleaacqWGPbqAaeqaaaqaaiabdMgaPbqab0GaeyyeIuoaaOGaayjkaiaawMcaaiabgkHiTmaabmaabaGae8hTdq2aaSbaaSqaaiabdsfaubqabaGccqGHRaWkdaaeqbqaaiab=j7aInaaBaaaleaacqWGRbWAaeqaaOGaemOvay1aaSbaaSqaaiabdUgaRbqabaaabaGaem4AaSgabeqdcqGHris5aaGccaGLOaGaayzkaaGaemivaq1aaSbaaSqaaiabdMgaPbqabaGccqGGSaalaaa@65C0@

I˙k=(∑k′μkk′βk′Vk′)(∑iTi)−(δI+∑iγki(I)Ti)Ik,
 MathType@MTEF@5@5@+=feaafiart1ev1aaatCvAUfKttLearuWrP9MDH5MBPbIqV92AaeXatLxBI9gBaebbnrfifHhDYfgasaacH8akY=wiFfYdH8Gipec8Eeeu0xXdbba9frFj0=OqFfea0dXdd9vqai=hGuQ8kuc9pgc9s8qqaq=dirpe0xb9q8qiLsFr0=vr0=vr0dc8meaabaqaciaacaGaaeqabaqabeGadaaakeaacuWGjbqsgaGaamaaBaaaleaacqWGRbWAaeqaaOGaeyypa0ZaaeWaaeaadaaeqbqaaGGaciab=X7aTnaaBaaaleaacqWGRbWAcuWGRbWAgaqbaaqabaGccqWFYoGydaWgaaWcbaGafm4AaSMbauaaaeqaaOGaemOvay1aaSbaaSqaaiqbdUgaRzaafaaabeaaaeaacuWGRbWAgaqbaaqab0GaeyyeIuoaaOGaayjkaiaawMcaamaabmaabaWaaabuaeaacqWGubavdaWgaaWcbaGaemyAaKgabeaaaeaacqWGPbqAaeqaniabggHiLdaakiaawIcacaGLPaaacqGHsisldaqadaqaaiab=r7aKnaaBaaaleaacqWGjbqsaeqaaOGaey4kaSYaaabuaeaacqWFZoWzdaqhaaWcbaGaem4AaSMaemyAaKgabaGaeiikaGIaemysaKKaeiykaKcaaOGaemivaq1aaSbaaSqaaiabdMgaPbqabaaabaGaemyAaKgabeqdcqGHris5aaGccaGLOaGaayzkaaGaemysaK0aaSbaaSqaaiabdUgaRbqabaGccqGGSaalaaa@5F73@

V˙k=πIk−(c+∑iγki(V)Ti)Vk.
 MathType@MTEF@5@5@+=feaafiart1ev1aaatCvAUfKttLearuWrP9MDH5MBPbIqV92AaeXatLxBI9gBaebbnrfifHhDYfgasaacH8akY=wiFfYdH8Gipec8Eeeu0xXdbba9frFj0=OqFfea0dXdd9vqai=hGuQ8kuc9pgc9s8qqaq=dirpe0xb9q8qiLsFr0=vr0=vr0dc8meaabaqaciaacaGaaeqabaqabeGadaaakeaacuWGwbGvgaGaamaaBaaaleaacqWGRbWAaeqaaOGaeyypa0dcciGae8hWdaNaemysaK0aaSbaaSqaaiabdUgaRbqabaGccqGHsisldaqadaqaaiabdogaJjabgUcaRmaaqafabaGae83SdC2aa0baaSqaaiabdUgaRjabdMgaPbqaaiabcIcaOiabdAfawjabcMcaPaaakiabdsfaunaaBaaaleaacqWGPbqAaeqaaaqaaiabdMgaPbqab0GaeyyeIuoaaOGaayjkaiaawMcaaiabdAfawnaaBaaaleaacqWGRbWAaeqaaOGaeiOla4caaa@4AFD@

The following cell types are considered: T-helper (CD4+) cells carrying the CCR5 co-receptor responding to virus strain *i*, (*T*_*i*_); T cells infected by virus strain *k*, (*I*_*k*_); *k *strains of R5 virus, (*V*_*k*_). We have thus assumed that viral strain *k *are identified by just one epitope, which is then displayed on the surface of the T cell of class *k*, and that a T cell of class *i *can be activated at least by one CD4+ T cell carrying the epitope *k*, which is specific of the viral strain *k*. The indices *i *(*k*) range from 1 to *N*_*i *_(*N*_*k*_), and in the following we have used *N*_*i *_= *N*_*k *_= *N*.

In Equation (4) T cells are generated through two mechanisms: the bone-marrow source (and selection in the thymus) and the duplication of T cell strains activated upon the recognition with an antigen carrying cell that may be even an infected one. We modeled T cells activation as a logistic term mimicking the global carrying capacity (the *K *parameter) of immune system [25].

The death-rate term is composed by a natural death rate proportional to the population, and by the infection rate of T cells due to any viral strain. The term ∑_*k*_*β*_*k*_*V*_*k*_*T*_*i *_and the sum over *T*_*i *_in the *I *cell birth rate reproduce the infection probability, that is the same irrespective of the T class. As a cell become infected it does no more contribute to the immune response.

Equation (5) describes the infection dynamics. The two death rate parameters account for the decrease of the infected cells due to cellular death and after the action of T-killer cells (CD8+). Even if there are clear experimental evidences that CD4+ cells decrease during the late HIV infection stages and in the AIDS state, as far as the asymptomatic phase of the infection is concerned, the parameter *δ*_*I *_may be assumed as a constant, medical literature referred, value.

The *μ*_*kk' *_term is responsible of the mutation process affecting the phenotype, essential for the formation of new quasispecies. The choice of a mutation rate of the order of 10^-5 ^is based on considering only those non-synonymous mutations that alter the phenotype (protein structure) [25].

In Equation (6) the virus replicative dynamics is described. The birth rate term is proportional to the virus "budding" numerosity while the viral death rate parameters depend on the rate of natural death and accounts for the recognition of virus by B cells.

It's worth noting that B-cells and T-killer cells are only implicitly included in the model in order to reduce the dimensionality without loosing too many details. We assume that these responses are fast enough to be at equilibrium and they are just proportional to the abundance of (cognate) *T *helper cells.

The three *γ *parameters *γ*^(*T*)^, *γ*^(*I*) ^and *γ*^(*V*) ^are matrices describing the interactions between cells and/or cells and viruses, i.e. who will interact with whom, in terms of geometry and strength of the interaction. It is thus possible to consider which strains of the virus are recognized and with which accuracy, and the same for the action of T-killer cells and B-cells. It is also worth noting that γii(T)
 MathType@MTEF@5@5@+=feaafiart1ev1aaatCvAUfKttLearuWrP9MDH5MBPbIqV92AaeXatLxBI9gBaebbnrfifHhDYfgasaacH8akY=wiFfYdH8Gipec8Eeeu0xXdbba9frFj0=OqFfea0dXdd9vqai=hGuQ8kuc9pgc9s8qqaq=dirpe0xb9q8qiLsFr0=vr0=vr0dc8meaabaqaciaacaGaaeqabaqabeGadaaakeaaiiGacqWFZoWzdaqhaaWcbaGaemyAaKMaemyAaKgabaGaeiikaGIaemivaqLaeiykaKcaaaaa@3420@ is the most important determinant of the viral fitness [25].

#### Modeling the transition R5 to X4

In about half of the people who develop advanced HIV disease, the virus begins to use another co-receptor called CXCR4 (X4 viral phenotype). The shift to using CXCR4 it is generally accompanied by a dramatic increase in the rate of T-cell depletion. The inability of the thymus to efficiently compensate for even a relatively small loss of naive T cells may be a key factor for CD4+ T cells depletion and AIDS progression. We hypothesize that it may not be exhaustion of homeostatic responses, but rather thymic homeostatic failure along with gradual wasting of T cell supplies through hyperactivation of the immune system that lead to CD4 depletion in HIV-1 infection.

We here introduce a modified version of the previous model, that considers only CD4 dynamics. Neither B nor CD8+ T cells are explicitly modeled and the space that is considered is that of different phenotypes of the virus. Under those assumptions we may focus on the appearance of X4 viruses and on their subsequent interaction with R5 strains.

dUdt=NU−δUU−δFUUF
 MathType@MTEF@5@5@+=feaafiart1ev1aaatCvAUfKttLearuWrP9MDH5MBPbIqV92AaeXatLxBI9gBaebbnrfifHhDYfgasaacH8akY=wiFfYdH8Gipec8Eeeu0xXdbba9frFj0=OqFfea0dXdd9vqai=hGuQ8kuc9pgc9s8qqaq=dirpe0xb9q8qiLsFr0=vr0=vr0dc8meaabaqaciaacaGaaeqabaqabeGadaaakeaadaWcaaqaaiabbsgaKjabdwfavbqaaiabbsgaKjabdsha0baacqGH9aqpcqWGobGtdaWgaaWcbaGaemyvaufabeaakiabgkHiTGGaciab=r7aKnaaCaaaleqabaGaemyvaufaaOGaemyvauLaeyOeI0Iae8hTdq2aa0baaSqaaiabdAeagbqaaiabdwfavbaakiabdwfavjabdAeagbaa@421C@

dTidt=δUU−(∑kβkVk)Ti−δTTi
 MathType@MTEF@5@5@+=feaafiart1ev1aaatCvAUfKttLearuWrP9MDH5MBPbIqV92AaeXatLxBI9gBaebbnrfifHhDYfgasaacH8akY=wiFfYdH8Gipec8Eeeu0xXdbba9frFj0=OqFfea0dXdd9vqai=hGuQ8kuc9pgc9s8qqaq=dirpe0xb9q8qiLsFr0=vr0=vr0dc8meaabaqaciaacaGaaeqabaqabeGadaaakeaadaWcaaqaaiabbsgaKjabdsfaunaaBaaaleaacqWGPbqAaeqaaaGcbaGaeeizaqMaemiDaqhaaiabg2da9GGaciab=r7aKnaaCaaaleqabaGaemyvaufaaOGaemyvauLaeyOeI0YaaeWaaeaadaaeqbqaaiab=j7aInaaBaaaleaacqWGRbWAaeqaaOGaemOvay1aaSbaaSqaaiabdUgaRbqabaaabaGaem4AaSgabeqdcqGHris5aaGccaGLOaGaayzkaaGaemivaq1aaSbaaSqaaiabdMgaPbqabaGccqGHsislcqWF0oazdaahaaWcbeqaaiabdsfaubaakiabdsfaunaaBaaaleaacqWGPbqAaeqaaaaa@4E33@

dIkdt=(∑k′μkk′βk′Vk′)(∑iTi)−δII
 MathType@MTEF@5@5@+=feaafiart1ev1aaatCvAUfKttLearuWrP9MDH5MBPbIqV92AaeXatLxBI9gBaebbnrfifHhDYfgasaacH8akY=wiFfYdH8Gipec8Eeeu0xXdbba9frFj0=OqFfea0dXdd9vqai=hGuQ8kuc9pgc9s8qqaq=dirpe0xb9q8qiLsFr0=vr0=vr0dc8meaabaqaciaacaGaaeqabaqabeGadaaakeaadaWcaaqaaiabbsgaKjabdMeajnaaBaaaleaacqWGRbWAaeqaaaGcbaGaeeizaqMaemiDaqhaaiabg2da9maabmaabaWaaabuaeaaiiGacqWF8oqBdaWgaaWcbaGaem4AaSMafm4AaSMbauaaaeqaaOGae8NSdi2aaSbaaSqaaiqbdUgaRzaafaaabeaakiabdAfawnaaBaaaleaacuWGRbWAgaqbaaqabaaabaGafm4AaSMbauaaaeqaniabggHiLdaakiaawIcacaGLPaaadaqadaqaamaaqafabaGaemivaq1aaSbaaSqaaiabdMgaPbqabaaabaGaemyAaKgabeqdcqGHris5aaGccaGLOaGaayzkaaGaeyOeI0Iae8hTdq2aaWbaaSqabeaacqWGjbqsaaGccqWGjbqsaaa@5115@

dVkdt=πIk−cVk
 MathType@MTEF@5@5@+=feaafiart1ev1aaatCvAUfKttLearuWrP9MDH5MBPbIqV92AaeXatLxBI9gBaebbnrfifHhDYfgasaacH8akY=wiFfYdH8Gipec8Eeeu0xXdbba9frFj0=OqFfea0dXdd9vqai=hGuQ8kuc9pgc9s8qqaq=dirpe0xb9q8qiLsFr0=vr0=vr0dc8meaabaqaciaacaGaaeqabaqabeGadaaakeaadaWcaaqaaiabbsgaKjabdAfawnaaBaaaleaacqWGRbWAaeqaaaGcbaGaeeizaqMaemiDaqhaaiabg2da9GGaciab=b8aWjabdMeajnaaBaaaleaacqWGRbWAaeqaaOGaeyOeI0Iaem4yamMaemOvay1aaSbaaSqaaiabdUgaRbqabaaaaa@3E0B@

dFdt=kF∑k∈X4Vk
 MathType@MTEF@5@5@+=feaafiart1ev1aaatCvAUfKttLearuWrP9MDH5MBPbIqV92AaeXatLxBI9gBaebbnrfifHhDYfgasaacH8akY=wiFfYdH8Gipec8Eeeu0xXdbba9frFj0=OqFfea0dXdd9vqai=hGuQ8kuc9pgc9s8qqaq=dirpe0xb9q8qiLsFr0=vr0=vr0dc8meaabaqaciaacaGaaeqabaqabeGadaaakeaadaWcaaqaaiabbsgaKjabdAeagbqaaiabbsgaKjabdsha0baacqGH9aqpcqWGRbWAdaWgaaWcbaGaemOrayeabeaakmaaqafabaGaemOvay1aaSbaaSqaaiabdUgaRbqabaaabaGaem4AaSMaeyicI4SaemiwaGLaeGinaqdabeqdcqGHris5aaaa@3F7A@

**Figure 2:**
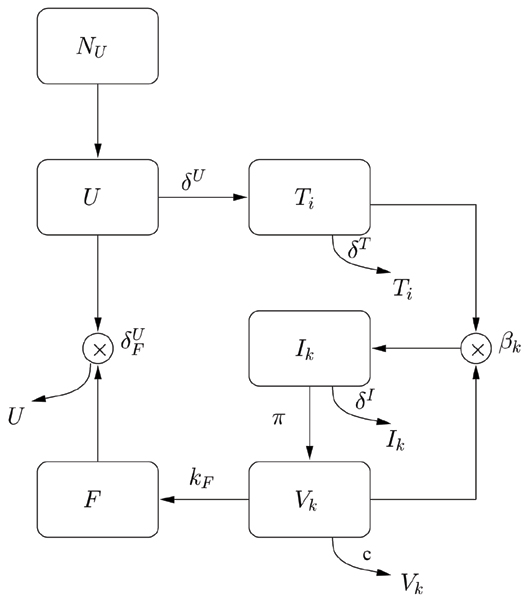
**Schematic description of the model for the switching from R5 to X4 viral phenotype**. Schematic description of the model for the switching from R5 to X4 viral phenotype. Naïve T-cells, *U*, are generated at constant rate *N*_*U *_and removed at rate *δ*^*U*^. They give birth to differentiated, uninfected T-cells, *T*. These in turn are removed at constant rate *δ*^*T *^and become infected as they interact with the virus. Infected T-cells, *I*, die at rate *δ*^*I *^and contribute to the budding of viral particles, *V*, that are cleared out at rate *c*. As soon as the X4 phenotype arise, the production of the TNF starts, proportional to the X4 concentration and contribute to the clearance of naïve T-cells, via the δFU
 MathType@MTEF@5@5@+=feaafiart1ev1aaatCvAUfKttLearuWrP9MDH5MBPbIqV92AaeXatLxBI9gBaebbnrfifHhDYfgasaacH8akY=wiFfYdH8Gipec8Eeeu0xXdbba9frFj0=OqFfea0dXdd9vqai=hGuQ8kuc9pgc9s8qqaq=dirpe0xb9q8qiLsFr0=vr0=vr0dc8meaabaqaciaacaGaaeqabaqabeGadaaakeaaiiGacqWF0oazdaqhaaWcbaGaemOrayeabaGaemyvaufaaaaa@30CD@ parameter.

In the equations above, the variables modeled are the pool of immature CD4+ T cells, *U*, the different strains of uninfected and infected T cells (*T *and *I*, respectively), HIV virus, *V*, and the excess of TNF concentration, *F*. TNF concentration, in uninfected people, i.e. when X4 = 0, has a low basal value. Here we consider the excess of TNF abundance, by assuming its concentration to be proportional to the X4 concentration. A schematic view of the model is depicted in Figure 2. The value of the parameters introduced with respect to the R5 model are summarized in Table 1.

**Table 1:** Additional parameters introduced in the R5 to X4 phenotypic switch model.

Parameter	Symbol	Value	Units of Meas.
Production of immature T cells	*N*_*U*_	100	cell/*μ*l *t*^-1^
Death rate of immature T cells	*δ*^*U*^	0.1	*t*^-1^
Death rate of immature T cells upon the interaction with TNF	δFU MathType@MTEF@5@5@+=feaafiart1ev1aaatCvAUfKttLearuWrP9MDH5MBPbIqV92AaeXatLxBI9gBaebbnrfifHhDYfgasaacH8akY=wiFfYdH8Gipec8Eeeu0xXdbba9frFj0=OqFfea0dXdd9vqai=hGuQ8kuc9pgc9s8qqaq=dirpe0xb9q8qiLsFr0=vr0=vr0dc8meaabaqaciaacaGaaeqabaqabeGadaaakeaaiiGacqWF0oazdaqhaaWcbaGaemOrayeabaGaemyvaufaaaaa@30CD@	10^-5^	*μ*l/cell *t*^-1^
Decreasing infectivity of R5 phenotype due to TNF	*k*_*R*5_	10^-7^	(*μl/*cell)^2 ^*t*^-1^
Increasing infectivity of R5 phenotype due to TNF	*k*_*X*4_	10^-7^	(*μl/*cell)^2 ^*t*^-1^
Increasing death rate of immature T cells due to TNF	δX4I MathType@MTEF@5@5@+=feaafiart1ev1aaatCvAUfKttLearuWrP9MDH5MBPbIqV92AaeXatLxBI9gBaebbnrfifHhDYfgasaacH8akY=wiFfYdH8Gipec8Eeeu0xXdbba9frFj0=OqFfea0dXdd9vqai=hGuQ8kuc9pgc9s8qqaq=dirpe0xb9q8qiLsFr0=vr0=vr0dc8meaabaqaciaacaGaaeqabaqabeGadaaakeaaiiGacqWF0oazdaqhaaWcbaGaemiwaGLaeGinaqdabaGaemysaKeaaaaa@31CF@	0.0005	*μ*l/cell *t*^-1^
Rate of production of TNF	*k*_*F*_	0.0001	*t*^-1^

Model for the R5 to X4 phenotypic switch: a summary of the additional parameters introduced. The value of the other parameters are medical literature referred, see also [39].

In particular, Equation (7) describes the constant production of immature T cells by the thymus *N*_*U *_and their differentiation into mature T cells at rate *δ*^*U*^. If X4 viruses are present, upon the interaction with TNF, immature T-cells are cleared at fixed rate δFU
 MathType@MTEF@5@5@+=feaafiart1ev1aaatCvAUfKttLearuWrP9MDH5MBPbIqV92AaeXatLxBI9gBaebbnrfifHhDYfgasaacH8akY=wiFfYdH8Gipec8Eeeu0xXdbba9frFj0=OqFfea0dXdd9vqai=hGuQ8kuc9pgc9s8qqaq=dirpe0xb9q8qiLsFr0=vr0=vr0dc8meaabaqaciaacaGaaeqabaqabeGadaaakeaaiiGacqWF0oazdaqhaaWcbaGaemOrayeabaGaemyvaufaaaaa@30CD@.

Equation (8) describes how uninfected mature T cells of strain *i *are produced at fixed rate *δ*^*U *^by the pool of immature T cells. Those cells, upon the interaction with any strain of the virus, *V*_*k*_, become infected at rate *β*_*k *_= *β *∀ *k*. The infectiousness parameter, *β*, is not constant over time, but depends on the interplay between R5 and X4 viruses. In particular, due to the increase in TNF abundance upon X4 emergence, the infectivity of R5 strains is reduced (*β*_*R*5_(*t*) = *β *- *k*_*R*5_*F*(*t*)), while the one of X4 viruses increases, with constant of proportionality *k*_*X*4 _(*β*_*X*4_(*t*) = *β *+ *k*_*X*4 _*F*(*t*)), mimicking the cell syncytium effect induced by the TNF molecule.

Equation (9) describes the infection of mature T-cells. Infected T-cells of strain *k *arise upon the interaction of a virus of strain *k *with any of the mature T-cell strains. The infected cells, in turn, are cleared out at a rate *δ*^*I*^. When TNF is released, this value increases linearly with constant δX4I
 MathType@MTEF@5@5@+=feaafiart1ev1aaatCvAUfKttLearuWrP9MDH5MBPbIqV92AaeXatLxBI9gBaebbnrfifHhDYfgasaacH8akY=wiFfYdH8Gipec8Eeeu0xXdbba9frFj0=OqFfea0dXdd9vqai=hGuQ8kuc9pgc9s8qqaq=dirpe0xb9q8qiLsFr0=vr0=vr0dc8meaabaqaciaacaGaaeqabaqabeGadaaakeaaiiGacqWF0oazdaqhaaWcbaGaemiwaGLaeGinaqdabaGaemysaKeaaaaa@31CF@, *δ*^*I*^(*t*) = *δ*^*I *^+ δX4I
 MathType@MTEF@5@5@+=feaafiart1ev1aaatCvAUfKttLearuWrP9MDH5MBPbIqV92AaeXatLxBI9gBaebbnrfifHhDYfgasaacH8akY=wiFfYdH8Gipec8Eeeu0xXdbba9frFj0=OqFfea0dXdd9vqai=hGuQ8kuc9pgc9s8qqaq=dirpe0xb9q8qiLsFr0=vr0=vr0dc8meaabaqaciaacaGaaeqabaqabeGadaaakeaaiiGacqWF0oazdaqhaaWcbaGaemiwaGLaeGinaqdabaGaemysaKeaaaaa@31CF@*F*(*t*).

Equation (10) is close to that in the R5 quasispecies model, a part from different viral phenotypes being here considered.

Finally, in Equation (11), we model the dynamics of accumulation of TNF by assuming the increase in TNF level to be proportional, via the constant *k*_*F*_, to the total concentration of X4 viruses present.

#### Investigating the mutational pathway from R5 to X4

In our model we represent the different phenotypes by using a linear strain space ordered in terms of phenotype similarity. Although we are aware of the several recent works on HIV mutational dynamics and phylogenetic assessments, we thought that a meaningful way to estimate the mutational pathways between R5 and X4 seen is to use phylogenetic inference on chemokine receptor families. The assessment of phylogenies using likelihood framework depends on the choice of an evolutionary model [26]. We computed the maximum likelihood (ML) analysis of the CRs data set under different models of evolution: [27], JTT [28], WAG [29]. We used these models considering the incorporation of the amino acid frequencies of the chemokine data sets, ('+*F *'), and the heterogeneity of the rates of evolution, implemented using a gamma distribution ('+Γ') [30,31].

### Results

We have first extended Perelson's standard model to incorporate different antigen recognition abilities by the immune system and coexistence dynamics of different R5 strains of HIV virus. Our approach is a mean field one, i.e. we investigate the average quantities of these molecular species [32-36]. Then, we have modified the model to describe features of the latent phase of the infection, i.e. the R5 to X4 switch and the hyperstimulation of the T cell precursors through the TNF. Finally we have analysed the results of the phylogenetic inference on chemokine receptors.

#### Amplification of R5 strains: mutation, co-infection and super-infection

Recent works have shown that HIV quasispecies may compete [22,37] and that persistence of the initial or ancestor quasispecies is a good indicator for disease progression [38]. Burch and Chao [39] have shown that the evolution of an RNA virus is determined by its mutational neighborhood. As the phenotype divergence among viral strains arises from differences in selection pressure, these differences may lead, for instance, to a higher infection rate. Since the competition is through the immune system response and given that the phase space of antigen recognition is not homogeneously covered [40], the HIV high mutation rate allows the quasispecies to find regions with weak immune response. This competition may lead to speciation of viral strains.

**Figure 3:**
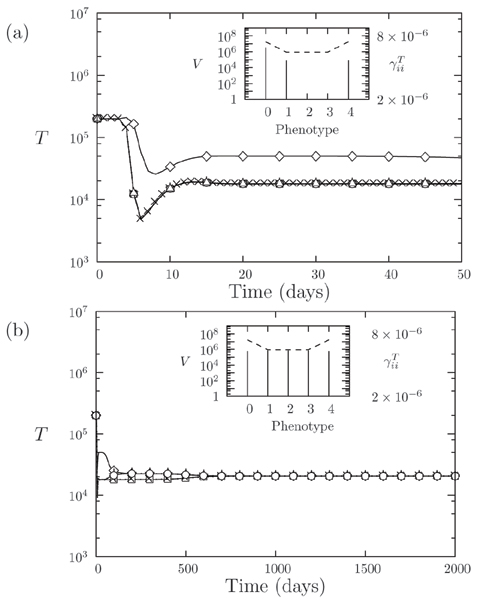
**Typical time evolution of the T cell abundance in the R5 model**. Typical time evolution of the T cell abundance in the R5 model for short-term (a) and long-term behavior (b). We set *μ *= 10^-5 ^with *N *= 5. In the inset, the y-axis on the left reports the abundance of virus strains at time *t *= 50 in plot (a) and *t *= 2000 in plot (b); the y-axis on the right shows the interaction strength (dashed line) between T cells and virus phenotypes (x-axis).

If we consider the model of the early phase of the infection, the evolution of T cells abundances in a scenario of quasispecies is shown in Figure 3a. Here we consider five viral phenotypes being differently recognized by the immune system. In particular, the recognition strength, corresponding to the selection pressure on viruses, is reported on the right y-axis in the inset of the figure as a dashed line. The temporal evolution of different T cells strains is characterized by the amplification of the T cells clones responding to viral infection, while asymptotic viral abundance is represented as solid stems in the inset. The asymptotic state of our model is a fixed point, thus the asymptotic distribution is insensitive of the initial conditions and the more abundant strains are that corresponding to the lower pressure exerted by the immune system (see inset of Figure 3b). However, one should consider that this asymptotic state may be reached after such a long time that it may be outside any practical scenario of the progression of a disease. The role of mutations in the transitory regime is quite particular. First of all, starting from the first inoculum at time *t *= 0 on the zeroth phenotype, mutations are necessary to populate the other strains of the virus, see also Figure 3. Moreover, in the presence of coupling among strains, due to competition or to a global constraints (the *K *parameter), the specific form of mutations does not play a fundamental role, see also Ref. [41].

**Figure 4:**
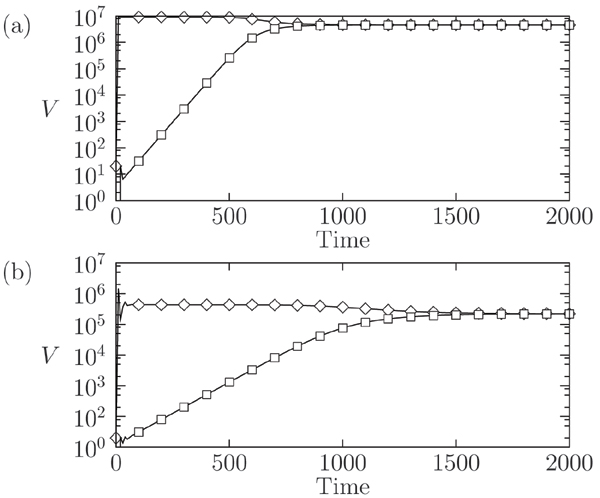
**Viral counts, *V*, during a superinfection scenario**. Viral counts, *V*, during a superinfection scenario. We set *N *= 5 and no mutation is considered, thus *μ *= 0. (a) A slow mounting of the second viral infection (□), having time scale of several months, is observed. In (b) a compromised immune system is considered. The time for the second strain to reach the same abundance of the first-infecting strain (◇) is greater than in (a).

Figures 4a and 4b show the results of short and long term viral coevolution after superinfection. We considered the first viral inoculum to happen at time *t *= 0, with the superinfection event occurring at time *t *= 20 days, when the immune response to the first inoculum has completed and the virus has established a chronic infection. After the second inoculum the model exhibits a short transient, followed by a slow mounting of the second infection. Due to the resulting low dynamics, the time needed by the second quasispecies to reach the same level of the other amounts to several months (Figure 4a) and represents another example of a slow relaxation toward a fixed-point equilibrium. We may also account for the progression of the disease by considering a weak immune system, for example being characterized by a lower thymic activity, i.e. a lower value of *λ*, with respect to the previous scenario. In this case the strain corresponding to the second inoculum requires much longer time to reach the same abundances of the first strain.

**Figure 5:**
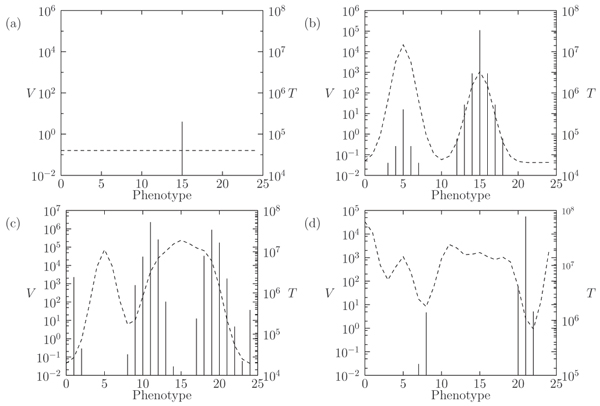
**Speciation of virus quasispecies**. Speciation of virus quasispecies and uninfected T cells dynamics after competitive superinfection at four different times: *t *= 0 (a), *t *= 4.5 (b), *t *= 5.25 (c) and *t *= 5.75 (d). Virus strain 15 is present at time *t *= 0, while strain 5 is inoculated at time *t *= 1. Mutation rate *μ *= 10^-4 ^and non-uniform interaction strength as in Figure 3. The dashed line represents the abundances of T cells targeting each viral phenotype, represented as vertical stems.

We have also investigated how the co-evolutionary and competitive dynamics of viral strains, mediated by the immune response, may lead to the formation of new viral quasispecies. We have considered a phenotypic space of 25 strains; this is an estimate of the number of different phenotypes (such those targeting different cell receptors or having different binding specifity for the same receptor) while the effective number of strains may be much larger. In Figure 5 the initial inoculum is at phenotype 15 (Figure 5a). In this simulation, since the immune system does not discriminate among similar phenotypes, there is an induced competition among neighboring strains. The result of this induced competition is the separation of the original quasispecies into two clusters (quasi-speciation), Figure 5b. However the immune system response continues to change in time (Figures 5c–d), resulting into a complex coevolution with viral populations.

#### R5 to X4 switch

Research into HIV dynamics has much to gain from investigating the evolution of chemokine co-receptor usage. Although CCR5 and CXCR4 are the major coreceptors used by HIV-1 a number of chemokine receptors display coreceptor activities in vitro. Several other chemokine receptors, possibly not present on the T cell membrane, may act as targets. To date, a number of human receptors, specific for these chemokine subfamilies, have been described, though many receptors are still unassigned. Several viruses, for example Epstein-Barr, Cytomegalovirus, and Herpes Samiri, contain functional homologous to human CRs, an indication that such viruses may use these receptors to subvert the effects of host chemokines [42]. Cells different from CD4 and CD8, such as macrophages, express lower levels of CD4, CCR5, and CXCR4 on the cell surface compared with CD4+ T cells [43-45], and low levels of these receptors expressed on macaque macrophages can restrict infection of some non-M-tropic R5 HIV-1 and X4 simian immunodeficiency virus (SIV) strains [46,47].

**Figure 6:**
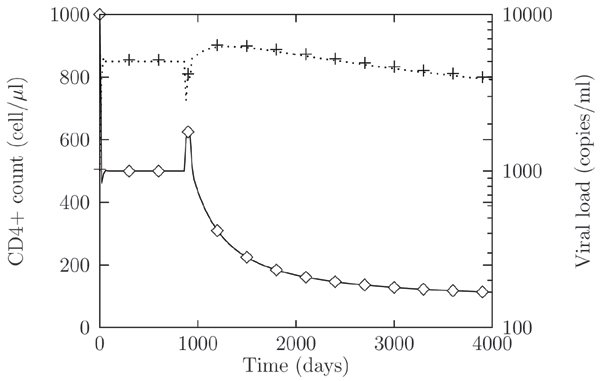
**R5 to X4 switch**. Time evolution of the concentrations of uninfected T-cells (◇) and viruses (+), during R5 to X4 switch, occurring at time *t *≈ 900. After the appearance of the X4 phenotype a continuous slow decline in CD4+ T-cells level leads to AIDS phase (CD4 counts below 200 cells/ml). We set *μ *≈ 0.001.

**Figure 7:**
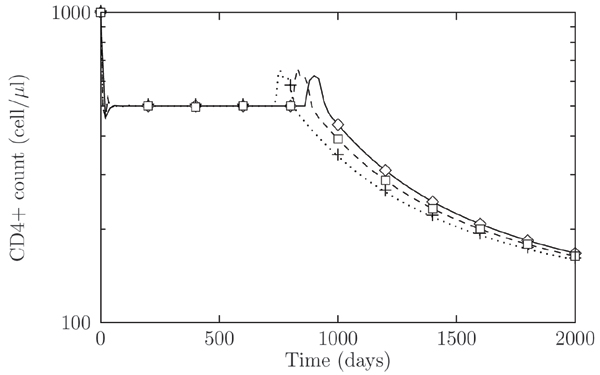
**CD4+ T-cells concentration during HIV-1 super-infection**. CD4+ T-cells concentration during HIV-1 super-infection by a R5 viral strain. Different signs represent: evolution without superinfection, (◇); superinfection occurring at time *t *= 100 and 400, (+) and (□), respectively. For a superinfection event occurring after the R5 to X4 switching the dynamics is qualitatively the same as for a single infection, (◇). If the second delayed infection occurs before the R5 to X4 switching, the time of appearance of X4 viruses may be shorter, when the superinfecting strain is closer to the X4 phenotypes, (+, □). Parameters as in Figure 6.

We studied the coevolutive dynamics leading to X4 strain appearance by successive mutations of the ancestor R5 strain. The stimulated production of TNF regulate the interactions between immune response and the virus and between the different strains of HIV virus. The results of these interactions are a decline in T-cells level, leading to the AIDS phase of the disease, and the decline in levels of viruses using the R5 coreceptor. In Figure 6 we report the result of the numerical simulations of the infection. The early stages of the infection are characterized by R5 strains. As time goes on, mutation accumulates that finally lead to the X4 phenotype appearance. After a short transitory regimes, T cells abundance starts declining. We have finally studied the influence of switching co-receptor usage in superinfection dynamics. In Figure 7 we show T-cells dynamics for different times of the superinfection event. We may observe that if the superinfection occurs after the appearance of the X4, the new R5 strain does not have any effect on T-cells behavior. On the other hand is worth noting that if the new R5 inoculum takes place before the X4 appearance, this may speed up the switching to the X4 phenotype if the new strain is mutationally close to the X4.

#### Investigating the mutational pathway between R5 and X4

Fundamental to the evolutionary approach is the representation of the evolution of sequences along lineages of evolutionary trees, as these trees describe the complex patterns of dependence amongst sequences that are caused by their common ancestry [29,48,49].

**Figure 8:**
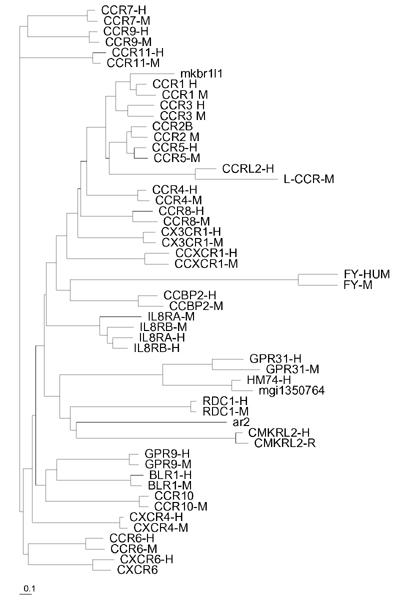
**Maximum likelihood phylogeny**. The maximum likelihood phylogeny under the JTT+F+Γ model of evolution for the set of human and mouse (mouse sequences are labelled with "-M") chemokine receptors. We have considered only the external loop regions. The scale bar refers to the branch lengths, measured in expected numbers of amino acid replacements per site.

The ML tree, obtained using the JTT+*F*+Γ model of evolution, is shown in Figure 8. The topology clearly shows that the CCR family is not homogeneous: CCR6, CCR7, CCR9 and CCR10 are separated from the other CCRs; in particular, CCR10 clusters with CXCRs; CXCR4 and CXCR6 do not cluster with the CXCRs. The tree shows that there are many mutational steps between CCR5 and CXCR4. The phylogeny suggests that the mutations that allow the virus env to cover a wide phenotypic distance from R5 to X4, may also lead to visit other receptors.

## Conclusion

The worldwide presence of several strains of the HIV virus and their often simultaneous presence within a patient, due to the increased frequency of multiple infections, are the remarkable features of HIV pandemia. For example, HIV-1 exists as several groups, subdivided into a growing number of subtypes which are slightly predominant in different geographical regions [50]. HIV-1-infected CD4+ T cells isolated from the spleens of two individuals were recently shown to harbor anywhere between one and eight proviruses, with an average of three to four proviruses per cell [51]. Mutations, recombination and selection pressure cause the appearance of quasispecies [52].

We first presented a model of the within-patience persistence of HIV quasispecies, by extending to multiple strain the Perelson's standard model [13]. This approach allows to incorporate coevolutive and competitive dynamics resulting from the different strains of HIV virus and different antigen recognition abilities by the immune system considered. Our model shows that the time evolution of the competition between quasispecies is slow and has time scales of several months.

Phylogenetic inference of chemokine receptors shows that there are several mutational patterns linking CCR5 to several receptors that have the same branch length of that from CCR5 to CXCR4. The evolutive dynamics towards the selection of X4 requires a preferential binding to a CXCR4 receptor. Recent works show that TNF is a prognostic marker for the progression of HIV disease [5,7] and has role in regulating the interactions between the different strains of HIV virus. The second model we have introduced shows that keeping low the concentration of TNF, both the depletion of T-cells precursors repertoire and the X4 dominance over R5 strains slow down.

The observed co-evolutionary dynamics of virus and immune response opens the way to the challenging possibility of the introduction or modulation of a quasispecies to be used in therapy against an already present aggressive strain, as experimented by Snell and colleagues [53,54]. Different drug treatments can alter the population of quasispecies. Will R5 blocking drugs cause HIV to start using X4? And will that be worse than letting the R5-using virus stay around along at its own, slower, but no less dangerous activity? The presence of a large number of R5 will increase the mutational spectra in R5 strains (late R5) and the probability of getting closer to the binding specificities of other chemokine receptors. Therefore, our model suggests that a therapy such the HAART to decrease the HIV load should be started the sooner the better. The large effect of TNF on T cells dynamics described by our model, suggests the benefit of a TNF buffering therapy. It is known that the dynamics of TNF is related to the dynamics of TNF-related apoptosis-inducing ligand (TRAIL). In this model we consider constant the concentration of TRAIL [7] and we do not consider many other important players such as Rantes.

Our models represent also a general framework to investigate intermittency or switching dominance of strains and the arising of new dominant strains during different phases of therapy; how superinfection will evolve in case of replacement of drug-resistant virus with a drug-sensitive virus and acquisition of highly divergent viruses of different strains; to investigate whether antiviral treatment may increase susceptibility to superinfection by decreasing antigen load.

## Competing interests

The authors declare that they have no competing interests.

## Authors' contributions

All authors conceived this project and drafted the manuscript. LS carried out most of simulations. All authors have interpreted and discussed the results, read and approved the final manuscript.
